# Learning to Read by Learning to Write: Evaluation of a Serious Game to Foster Business Process Model Comprehension

**DOI:** 10.2196/15374

**Published:** 2020-01-09

**Authors:** Michael Winter, Rüdiger Pryss, Thomas Probst, Manfred Reichert

**Affiliations:** 1 Institute of Databases and Information Systems Ulm University Ulm Germany; 2 Department for Psychotherapy and Biopsychosocial Health Danube University Krems Krems Austria

**Keywords:** business process model comprehension, business process modeling, serious games, learning, research design

## Abstract

**Background:**

The management and comprehension of business process models are of utmost importance for almost any enterprise. To foster the comprehension of such models, this paper has incorporated the idea of a serious game called Tales of Knightly Process.

**Objective:**

This study aimed to investigate whether the serious game has a positive, immediate, and follow-up impact on process model comprehension.

**Methods:**

A total of two studies with 81 and 64 participants each were conducted. Within the two studies, participants were assigned to a game group and a control group (ie, study 1), and a follow-up game group and a follow-up control group (ie, study 2). A total of four weeks separated study 1 and study 2. In both studies, participants had to answer ten comprehension questions on five different process models. Note that, in study 1, participants in the game group played the serious game before they answered the comprehension questions to evaluate the impact of the game on process model comprehension.

**Results:**

In study 1, inferential statistics (analysis of variance) revealed that participants in the game group showed a better immediate performance compared to control group participants (*P*<.001). A Hedges g of 0.77 also indicated a medium to large effect size. In study 2, follow-up game group participants showed a better performance compared to participants from the follow-up control group (*P*=.01); here, a Hedges g of 0.82 implied a large effect size. Finally, in both studies, analyses indicated that complex process models are more difficult to comprehend (study 1: *P*<.001; study 2: *P*<.001).

**Conclusions:**

Participants who played the serious game showed better performance in the comprehension of process models when comparing both studies.

## Introduction

### Background

The application of game designs and their related principles constitutes a promising approach to encouraging learning and to playfully imparting knowledge [1,2]. More specifically, this could mean integrating a game design and its principles into a nongame context (eg, administrative work). Among others, serious games have received attention as a potential alternative for fostering professional development by stimulating an active learning process [3]. By using specific design principles derived from video games (eg, competition, curiosity, and collaboration), serious games integrate these into a nongame context to improve motivation when completing or addressing complex or bothersome tasks [4]. Accordingly, there have been reviews and studies that investigated the potential impact of such games on learning and skill enhancement [5]. For example, Wouters et al [6] evaluated several game features (eg, role play) and outlined that serious games and their respective features can improve cognitive skills (eg, problem-solving). Furthermore, Von Wangenheim and Shull [7] demonstrated that serious games are an effective approach for learning, especially for the reinforcement of knowledge.

Owing to the increasing positive awareness of serious games, various disciplines (eg, education, health care, and business) have adapted the use of serious games according to their purposes [8-10]. Based on this, the computer science discipline that deals with the creation and understanding of process models is also suitable for the utilization of serious games [11]. A process model is a type of diagram that represents the procedures, workflows, and algorithms [12], but it specifically documents all steps, decisions, and involved persons needed to achieve a specific goal. Therefore, process models have been widely adopted in different domains (eg, computer science, and health care) [13,14]. In this context, business process models constitute an extension of process models, which are predominantly used in the field of business and industry for the documentation of respective business processes [15]. The creation of business process models (ie, business process modeling) and understanding them (ie, process model comprehension) are essential factors for enterprises to capture and work with their business processes daily [16]. In this context, the modeling and comprehension of processes and respective models are demanding tasks. Consequently, knowledge about the process to be modeled/understood, as well as respective expertise, is required to be able to use the advantages of business process models [17].

Knowledge in process modeling and the expertise to properly comprehend a process model are often acquired through work experience, formal training, or through educational institutions (eg, universities). Therefore, a proper education or training in process modeling must be ensured to understand how to model business processes correctly and, thus aim to create high-quality process models [18]. Furthermore, the capability to properly understand process models is accompanied by learning how to both create and model them. Vice versa, the inverse is only conditionally applicable; that is, learning to comprehend process models only fosters the ability to model processes to a limited extent [19].

In the context of our research, to foster the comprehension of process models, the application of serious games for training for process modeling and understanding offers promising opportunities to significantly improve them [20,21]. For process modeling and process model comprehension, specific research exists evaluating the potential use of serious games in this context. For example, a serious game approach with an emphasis on process enactment and a discussion about opportunities in the context of business process management has been elaborated on in a study by Herzberg and Kunze [22]. Vuksic and Bach [23] also gave suggestions about introducing a simulation game environment to foster an overall understanding of using business processes. Based on virtual environments, Ribeiro et al [24] introduced a serious game for teaching business process modeling, model comprehension, and process simulation. In turn, in a study by Aysolmaz et al [25], an office environment was virtualized using a three-dimensional virtual world to enable an immersive experience for improving process model comprehension. The results and insights obtained from a field study in which a serious game based on a business process model is used to familiarize employees with a complex process of a manufacturer were discussed in a study by Rosenthal and Strecker [26]. Finally, Mendling et al [27] discussed viewing characteristics of process modelers (eg, level of theoretical knowledge of modeling). Afterward, the characteristics that influence process model comprehension were investigated by taking gamification into account.

Beside expertise gained in process modeling and comprehension during the accomplishment of practical tasks, interestingly, many business analysts or process modelers learn more about business process modeling and their importance for enterprises in tertiary institutions (eg, universities) [28]. Nowadays, well-trained process modelers are crucial for enterprises to overcome many daily challenges. Understanding these models is also of utmost importance, as, for example, people need to know about their responsibilities and where to get relevant information from [29]. For example, enterprises must flexibly react to changes in the market. Therefore, they need to be able to align their business processes accordingly to satisfy technological and environmental evolutions or constraints. Thus, ensuring proper comprehension of process models through good education in the business process field should be a key factor for enterprises.

### Objective

To address this and enhance our understanding of process model comprehension, this paper presents a serious game to investigate its impact on the comprehension of process models. In particular, the presented serious game teaches the essentials of the business process modeling standard Business Process Model and Notation (BPMN) version 2.0 [30] to foster the general comprehension of process models. Furthermore, it must be noted that the objective of the serious game is not conveying the subtleties of BPMN version 2.0 but rather creating a fundamental understanding of working with process models. Therefore, the serious game introduces core concepts for process modeling. It also teaches the most common modeling elements of BPMN version 2.0 and their precise meaning for the creation of proper process models, including correct comprehension of the used modeling elements. Overall, to evaluate the impact of the serious game on process model comprehension, the following two research questions are addressed in this study: (1) is the comprehension of process models directly after playing the serious game better in participants playing the game than in those who did not play, and does this depend on the model complexity; and (2) is the comprehension of process models four weeks after playing the serious game better in participants who played than in those who did not and does this depend on the model complexity?

Two studies were conducted to investigate the raised questions. For question 1, process model comprehension of the participants who played the serious game was compared to the performance of those who did not play the serious game. For question 2, exactly four weeks after conducting the first study, the performances of the same participants who played the serious game during study 2 but not in study 1 were compared with each other. Therefore, question 2 was concerned with whether the serious game fostered comprehension of process models at the follow-up study. Finally, for both questions, the level of process model complexity was considered to evaluate whether the learning process is affected by the complexity of the process models.

## Methods

### Tales of a Knightly Process

The serious game, Tales of a Knightly Process, tells the story of King Rex, the ruler of the kingdom Processia. Unfortunately, via a raven messenger, the king gets to know that his future princess, Calidia, was abducted by the Black Knight. With all his fervor, King Rex mobilizes all his troops to release his beloved princess from the Black Knight’s claws.

The story of the serious game is divided into 3 acts, including a separate prologue and epilogue. Each act contains a set of levels in which a process model (expressed in terms of the BPMN version 2.0) has to be modeled for a specific situation (eg, a siege of the Black Knight’s castle). In total, 13 processes have to be modeled as a BPMN version 2.0 process model while playing the serious game. In the beginning, simple processes have to be modeled, but as one progresses, the processes become more and more complex. In the first half of the serious game, basic process modeling elements (eg, activities) and constructs (eg, loops) of BPMN version 2.0 are introduced and explained via narration by the king. In the second half of the serious game, the previously learned aspects of BPMN version 2.0 and process modeling are mainly repeated. Furthermore, in the first half of the game, the modeling of processes is the main focus, while in the second half of the game, there is an emphasis on process model comprehension. For a better overview, the BPMN version 2.0 elements used in the game are explained:

- Activities: An activity is an atomic or nonatomic task and describes an executable step in a process (eg, place order).
- Event: An event indicates that something is happening in the process which affects its flow (eg, error detected).
- Gateway: A gateway allows for control as well branching, and merges the process flow (eg, decision points).
- Sequence flows: Sequence flows connect all elements in a process model and represent the direction of the flow (eg, choreography).
- Pool: A pool describes an independent (organizational) unit with clearly defined boundaries in a process (eg, enterprise).
- Lane: A lane represents concrete people, roles, or departments within a pool (eg, sales department).
- Subprocess: A subprocess allows the modularization of (complex) process models into smaller models to reduce the complexity and increase comprehensibility.

### Serious Game Mechanics

**Figure 1:**
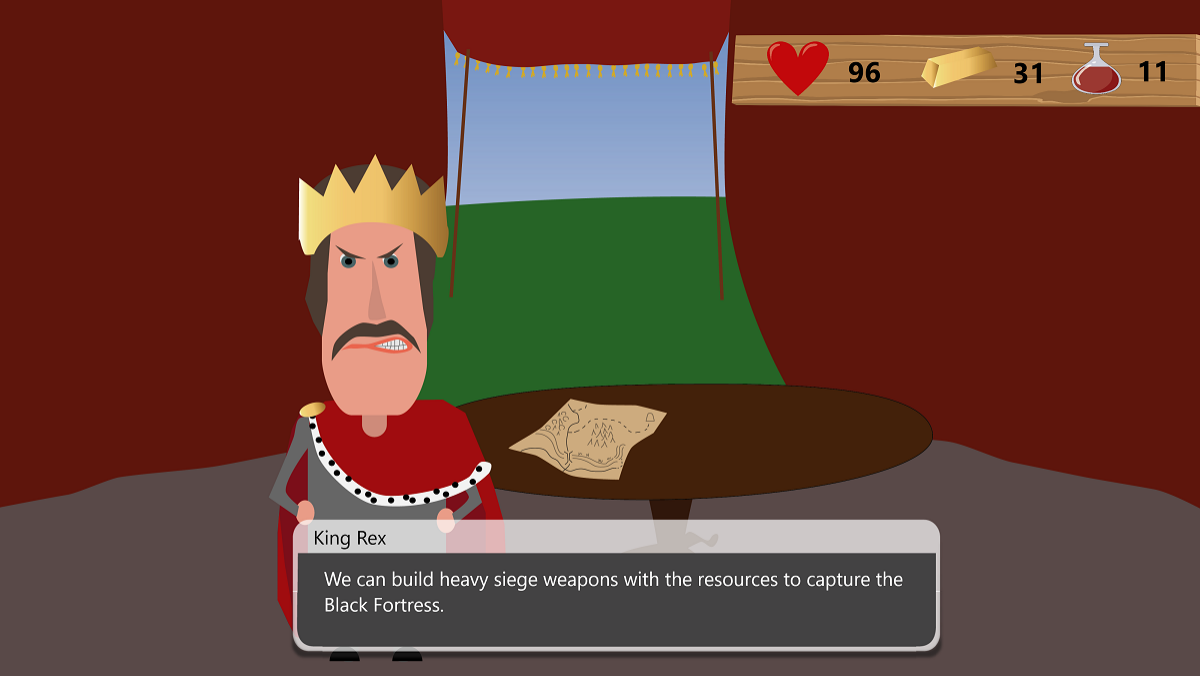
Storytelling of the serious game.

The story is told with the use of dialogue from the perspective of King Rex (see Figure 1). To save his princess, the king must reach the Black Knight’s fortress. On his way, he must prepare his troops, gather resources, and overcome dangers. Thus, these situations must be modeled in the serious game in terms of BPMN version 2.0 process models.

**Figure 2:**
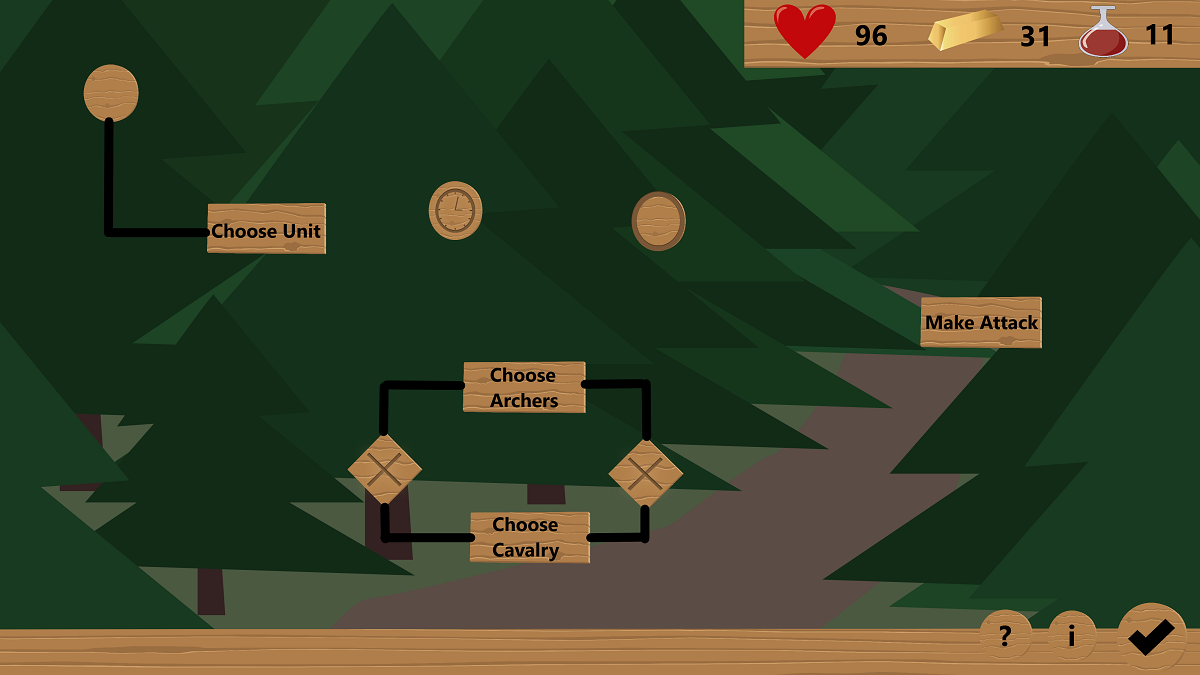
Process modeling environment.

To continue the story, a process model must be created for each level, which is done using a separate process modeling environment (see Figure 2). In this environment, only specific elements are available, all of which must be used to complete the level. Therefore, these particular elements must be placed and connected as specified in BPMN version 2.0. After a process model has been created, it is checked to determine whether it is correct. Progress to the next level is made only with a correct process model. Otherwise, the process model must be adapted accordingly.

**Figure 3:**
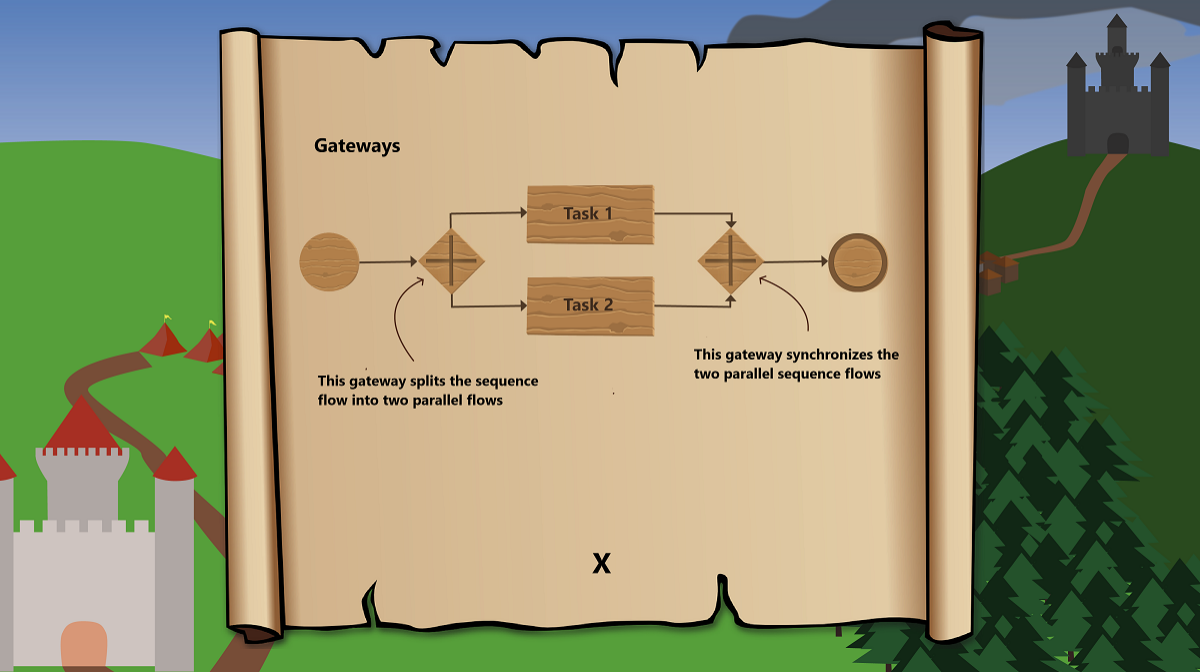
Description and help text.

**Figure 4:**
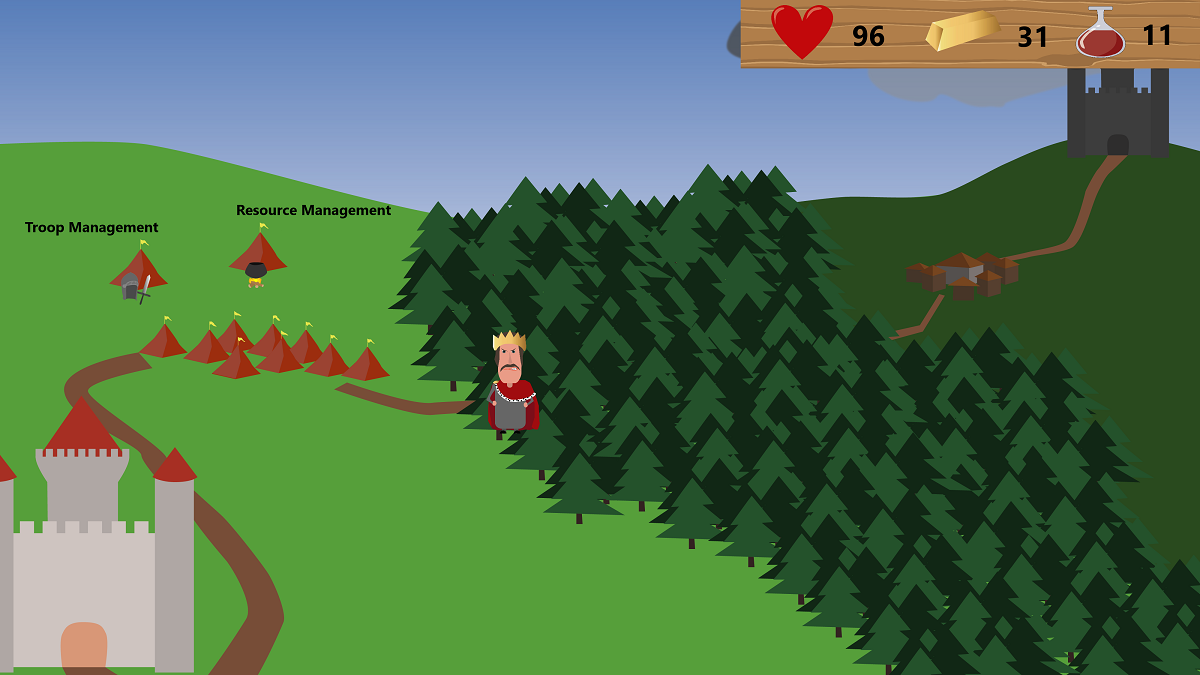
Troop and resource management.

For further assistance during process modeling, the serious game provides scrolls that contain hints and additional information about previously learned BPMN elements and constructs (see Figure 3). Specifically, the use of elements and their respective meanings are described in more detail alongside an example of their use. The main focus of the serious game is on the imparting of knowledge about BPMN version 2.0 and how to use it for process modeling, with an addition goal of fostering the comprehension of process models. However, the serious game also emphasizes the added value of curiosity and competition to hopefully induce higher motivation and an enhancement of the learning process. Therefore, the serious game offers additional concepts from video games, such as resource management and item crafting (see Figure 4). Furthermore, the serious game is designed to have a supporting narrative storyline, as the story is tailored by the choices made therein, thus increasing replayability.

### Comprehension Questions

For the evaluation of the impact of the serious game (ie, immediate [study 1] and follow-up [study 2]), all participants in both studies had to answer ten true or false comprehension questions on five different process models in a separate questionnaire. Specifically, two comprehension questions were asked per process model, which were expressed in terms of BPMN version 2.0. The five process models represented five different levels of complexity (ie, beginner, basic, intermediate, advanced, and expert).

The beginner process model was only composed of basic modeling elements. With the rising level of complexity, new BPMN version 2.0 elements were added, and the total number of elements was increased. The guidelines from studies by Becker et al and Mendling et al [31,32] as well as an adopted cognitive complexity measure proposed in a study by Gruhn and Laue [33], were used in the creation of the process models to allow categorization into these five levels of complexity. The process models documented no concrete scenario, and single alphabetic letters were used to label the process model elements (ie, abstract labeling type). The ten comprehension questions referred to the syntactic and semantic dimension of the process models and were used to evaluate the impact (ie, immediate and follow-up) of the serious game. The comprehension questions were defined by two process modeling experts in a consensus-building process.

In study 1, both groups received the same questionnaire with the same ten comprehension questions. In study 2, to ensure comparability between both studies, a similar but still different set of process models and related comprehension questions were used. More specifically, a small change was made in the structure of the process models and respective element labeling. For the comprehension questions, different questions were used, but they referred further to the syntactic and semantic dimensions of the process models.

### Participants

In study 1, the sample size comprised 81 students from an entry course in Business Process Management at Ulm University. Until the beginning of study 1, the entry course introduced the fundamentals of business process modeling to the students.

Overall, 36 participants were female and the average age was 23.67 years (SD 3.19). Based on a demographic questionnaire, 47 participants stated they had little to no experience in process modeling and comprehension as well as in working with BPMN version 2.0 (ie, 0), while 20 participants had average experience (ie, 1), and 14 participants had a high level of experience (ie, 2). The participants were divided into two groups (ie, control group and game group) using the round-robin approach (ie, alternating assignment into one of the two groups). Moreover, the round-robin approach ensured that both groups had similar characteristics in terms of baseline variables. Due to restrictions on the availability of mobile devices, 3 participants who belonged to the game group had to be assigned to the control group instead. In total, the control group consisted of 44 participants and the game group consisted of 37 participants. The baseline comparisons between the control and the game group participants are presented in Table 1. *P* values presented were calculated using the Fisher exact test.

In study 2, 64 students were enrolled from the same Business Process Management entry course at Ulm University. During the period between study 1 and study 2, the entry course taught the process modeling aspects that are included in the serious game.. For this reason, a general increase in the comprehension question scores was expected in study 2. All participants were divided into a follow-up control group and a follow-up game group. Specifically, 30 participants who played the serious game in study 1 (ie, identified by a generated pseudocode) were allocated to the follow-up game group [ie, FU_Game]. However, seven game group participants from study 1 did not participate in study 2 because of term fluctuation. A total of 34 participants who either participated in study 1 and did not play the game (n=31) and new participants who were not part of study 1 (n=3) were allocated to the follow-up control group [ie, FU_Control]. A total of 13 control group participants from study 1 did not participate in study 2 again because of term fluctuation. Overall, 27 females participated in study 2, and the average age was 23.50 years (SD 3.18). From the demographic questionnaire, 37 participants with little to no experience, 19 participants with average experience, and 8 participants with high experience in process modeling and comprehension took part in the study. The control and game group participants were compared along baseline variables, and the results are presented in Table 2. *P* values presented were calculated using the Fisher exact test.

**Table 1:** Sample description and comparison of study 1 in baseline variables.

Variables		Control (n=44)	Game (n=37)	*P* value
**Sex, n (%)**				.65
	Female	21 (52)	15 (41)	
	Male	23 (48)	22 (59)	
Average age (years), mean (SD)		23.23 (3.09)	24.20 (3.25)	
**Age, n (%)**				.24
	Aged <25 years	32 (73)	22 (59)	
	Aged >24 years	12 (27)	15 (41)	
**Experience, n (%)**				>.99
	0 (none to little)	26 (59)	21 (57)	
	1 (average)	11 (25)	9 (24)	
	2 (high)	7 (16)	7 (19)	

**Table 2:** Sample description and comparison of study 2 in baseline variables.

Variables		FU_Control (n=34)	FU_Game (n=30)	*P* value
**Sex, n (%)**				.45
	Female	16 (47)	11 (37)	
	Male	18 (53)	19 (63)	
Average age (years), mean (SD)		23.15 (3.09)	23.90 (3.24)	
**Age, n (%)**				.43
	Aged <25 years	25 (73)	19 (63)	
	Aged >24 years	9 (27)	11 (37)	
**Experience, n (%)**				>.99
	0 (none to little)	20 (59)	17 (57)	
	1 (average)	10 (29)	9 (30)	
	2 (high)	4 (12)	4 (13)	

### Comprehension Questions Score

To investigate the immediate and follow-up impact of the serious game, participants were asked to answer ten true or false comprehension questions on five different process models (ie, two questions each). The process models were categorized into five different levels of complexity, and five questions had to be answered per process model. The sum of the correct answers was used as the respective performance measure in study 1 and study 2.

### Study Design

The design of the study is based on the guidelines set out by Wohlin et al [34]. Before conducting the two studies, a pilot study of the serious game was performed that involved three participants who did a playtest with a think-aloud protocol. All three participants had experience with process modeling and comprehension as well as with BPMN version 2.0. The playtest ensured that no severe bugs or design flaws were in the final game. Think-aloud protocols support the identification of misunderstandings and usability problems that can be addressed accordingly. Following this, several quality improvements were implemented in the game’s final version, which included, among others, the improvement of the use of the single BPMN version 2.0 elements and the process models that needed to be modeled. Also, apart from the perceived fun factor, the educational emphasis of introducing BPMN version 2.0 and corresponding elements was discernible. Furthermore, for the evaluation of the immediate and follow-up impact of the serious game on process model comprehension, a longitudinal study design with two measurement time points was chosen. The time between the two measurements was four weeks.

The procedure of study 1 (ie, focus on the immediate impact of the serious game) was as follows: the study participants were welcomed, and the overall study procedure was explained. Following this, the participants were divided according to the round-robin approach (ie, alternating assignment into one of the two groups) into a control group and game group. After this step, study materials (ie, study description, demographic questionnaire, and study trial) were handed out to the participants. In the control group, first, the study description had to be read. Then, the participants needed to answer the demographic questionnaire to capture relevant demographic data (eg, age and gender).

After the demographic questionnaire was answered, participants had to answer ten true or false comprehension questions on five differently complex (ie, beginner, basic, intermediate, advanced, and expert) process models (ie, two comprehension questions per process model). The comprehension questions solely referred to the syntactical rules or the semantic description of the process models. After completing this step, the study ended. Thus, the control group did not play the serious game and only answered the comprehension questions. The game group, in turn, played the serious game on mobile devices after answering the demographic questionnaire to evaluate the immediate impact thereof. The serious game was played only once, with a playthrough taking about 20 minutes to complete. After playing the serious game, ten true or false comprehension questions (ie, the same as in the control group) on five differently complex process models had to be answered.

**Figure 5:**
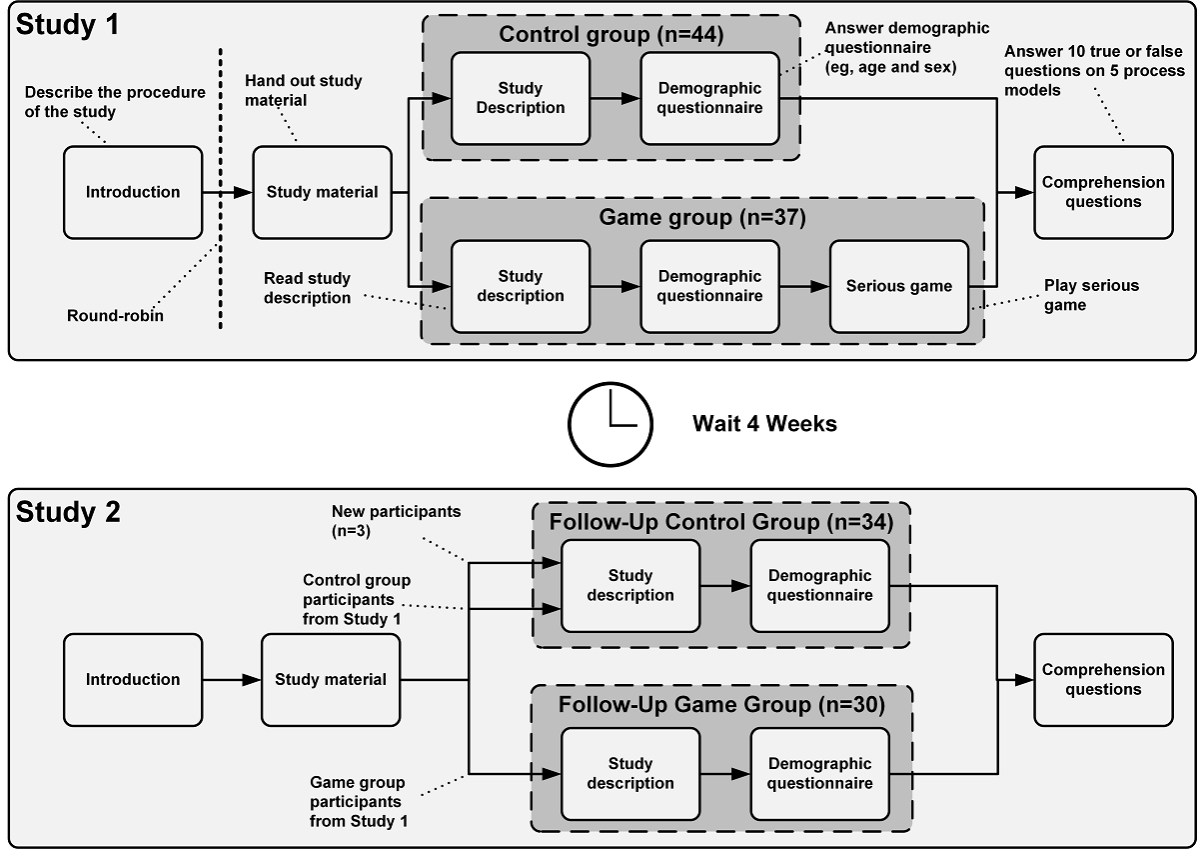
Study design.

In study 2 (ie, focus on the follow-up impact of the serious game), four weeks after study 1, participants were identified by a generated pseudocode based on if they had played the serious game in study 1 or not. Those who played the serious game in study 1 made up the follow-up game group, and those who did not play the game (either control group participants in study 1 or new participants) were the follow-up control group. The serious game was not played again in study 2. Afterward, all participants had to answer ten true or false comprehension questions on five process models of varying complexity. The comprehension questions and respective process models were like the ones used in study 1 to ensure comparability between the studies. The entire study design for study 1 and study 2 is outlined in Figure 5. During the four weeks between the two measurements (ie, study 1 and study 2), the entry course on Business Process Management introduced the aspects of BPMN version 2.0 used in the game to the participants as part of the syllabus. For this reason, we expected an increase in the comprehension question score in study 2 compared to study 1 for both groups.

## Results

### Descriptive Statistics

Regarding study 1, Table 3 presents descriptive results obtained from the control group and game group immediate after study 1. It shows the average correct answers per complexity level as well as the sum thereof. As can be seen from Table 3, participants who played the serious game (ie, game group) achieved a better result in the comprehension questions compared with participants who did not play the serious game (ie, control group). In general, the control group achieved a mean of 5.70 (SD 1.46), whereas the game group achieved a mean of 6.86 (SD 1.55). Furthermore, a decrease in the score is noticeable in both groups with rising level of process model complexity.

Regarding study 2, Table 4 shows descriptive results from both groups (ie, follow-up control group and follow-up game group). Once again, the table shows the average correct answers per complexity level and the sum thereof for the ten true or false questions that were asked. In general, there is an increase in the comprehension question score between study 1 and study 2. This general increase is explained by the fact that participants spent considerable time working with process models as well as BPMN version 2.0 in the context of the entry course. However, comparing the results from the follow-up control group and follow-up game group, the same observation as seen in study 1 is discernible. Specifically, after four weeks, participants who were in the game group in study 1 achieved a better result at answering comprehension questions compared to participants who did not play the serious game. The participants of the follow-up control group achieved a mean comprehension score of 6.65 (SD 1.63), while the follow-up game group achieved a mean score of 7.90 (SD 1.37).

**Table 3:** Descriptive results from study 1.

Group	Level of complexity (score), mean (SD)					
	Beginner	Basic	Intermediate	Advanced	Expert	Sum
Game (n=37)	1.68 (0.53)	1.59 (0.55)	1.41 (0.64)	1.16 (0.65)	1.03 (0.76)	6.86 (1.55)
Control (n=44)	1.55 (0.59)	1.23 (0.71)	1.23 (0.68)	0.95 (.53)	0.75 (.61)	5.70 (1.46)

**Table 4:** Descriptive results from study 2.

Group (n)	Level of complexity (score), mean (SD)					
	Beginner	Basic	Intermediate	Advanced	Expert	Sum
FU_Game (n=30)	1.87 (0.35)	1.73 (0.52)	1.57 (0.50)	1.57 (0.50)	1.17 (0.70)	7.90 (1.37)
FU_Control (n=34)	1.61 (0.49)	1.44 (0.70)	1.38 (0.60)	1.24 (0.74)	0.97 (0.67)	6.65 (1.63)

### Inferential Statistics

To evaluate the reported descriptive results for statistical significance, analyses of variance for repeated measurements were performed with the performance measure score as dependent variable (Greenhouse-Geisser correction was applied). For these analyses, the within-subject factor had five levels and was the level of process model complexity, and the between-subject factor had two levels and consisted, for all research questions, of the sample comparison of interest (ie, control vs game and follow-up control vs follow-up game). The main effects of the level of complexity and sample comparison, as well as the interaction effect (ie, complexity×sample comparison), were evaluated. In addition, in the event of significance for the level of complexity and the interaction effect, repeated contrasts were employed. Finally, for question 1 and questions 2, Hedges g was calculated to quantify the effect of the serious game. Effect sizes will be interpreted as follows: 0.20=small effect, 0.50=medium effect, and 0.80=large effect. All two-tailed statistical tests were performed, and the significance value was set to *P*<*.*05.

### Results for Research Question 1

Table 5 presents the results for the performance measure score for research question 1.

Main effect 1, the level of complexity, was significant (*P*<.001), and repeated contrasts showed that complexity 2 (*P*=.04) had a lower score (mean 1.40; SD 0.66) than complexity 1 (mean 1.60; SD 0.56) and, in addition, complexity 3 (*P*=.36) did not have a lower score (mean 1.31; SD 0.66) than complexity 2. However, complexity 4 (*P*=.02) had a lower score (mean 1.05; SD 0.59) than complexity 3, whereas complexity 5 (*P*=.08) did not have a lower score (mean 0.88; SD 0.70) than complexity 4. Furthermore, main effect 2, the sample comparison, reached statistical significance (*P*<.001), and the control group had significantly lower scores than the game group. The interaction effect did not reach significance. Finally, a Hedges g was determined for each level of complexity and the sum thereof as between-group effect size. Hedges g for beginner, basic, intermediate, advanced, and expert was 0.23, 0.56, 0.27, 0.35, and 0.41, respectively, and Hedges g for sum was 0.77.

**Table 5:** Inferential statistics for research question 1.

Effect	Performance measure score	
	*F* test (df)	*P* value
Main effect of the level of complexity	17.19 (3.68, 290.50)	<.001
Main effect of sample comparison	12.05 (1, 79)	<.001
Interaction effect	0.45 (3.68, 290.50)	.76

### Results for Research Question 2

Table 6 shows the results for the performance measure score with respect to research question 2.

Main effect 1, the level of complexity, was significant (*P*<.001), and repeated contrasts showed that complexity 2 (*P*=.11) did not have a lower score (mean 1.58; SD 0.64) than complexity 1 (mean 1.74; SD 0.45) and, in addition, complexity 3 (*P*=.26) did not have a lower score (mean 1.47; SD 0.56) than complexity 2. Furthermore, complexity 4 (*P*=.50) did not have a lower score (mean 1.39; SD 0.66) than complexity 3, whereas complexity 5 (*P*=.002) had a lower score (mean 1.06; SD 0.69) than complexity 4. Moreover, main effect 2, the sample comparison, reached statistical significance (*P*=.01), and the follow-up control group had significantly lower scores than the follow-up game group. Furthermore, the interaction effect did not attain significance. Finally, a Hedges g was determined for each level of complexity and the sum thereof as a between-group effect size. Hedges g for beginner, basic, intermediate, advanced, and expert was 0.60, 0.46, 0.34, 0.51, and 0.29, respectively, and Hedges g for sum was 0.82.

**Table 6:** Inferential statistics for research question 2.

Effect	Performance measure score	
	*F* test (df)	*P* value
Main effect of the level of complexity	12.28 (3.81, 236.45)	*<*.001
Main effect of sample comparison	10.89 (1, 62)	.01
Interaction effect	0.19 (3.81, 236.45)	.94

## Discussion

### Principal Findings

This study evaluated a serious game to foster business model comprehension. First, we evaluated whether participants who played the serious game showed a better immediate performance than participants who did not play the serious game, and also whether this depended on the level of complexity of the process models (ie, research question 1). We found that participants who played the serious game had a significantly better immediate performance measure than participants who did not play the serious game and that this difference between the game group and control group was not affected by process model complexity. The corresponding between-group effect size was medium to large for the sum score of model comprehension (g=0.77). Based on the presented results, it can be concluded that the serious game had a significant, positive, immediate, impact on process model comprehension. Moreover, in this scenario, the comprehension of process models is not improved through direct approaches focusing on respective comprehensibility. In turn, with an indirect approach about how to model processes, an improvement in process model comprehension was addressed.

Second, we analyzed whether participants who played the serious game showed a better follow-up performance measure than participants who did not play the serious game. It was again analyzed whether this depended on the level of complexity of the process models. We found that participants who played the serious game had a significantly better follow-up performance measure than participants who did not play the serious game and that this group difference was not affected by the process model complexity. Furthermore, Hedges g implied a large effect for the sum score of model comprehension (g=0.82). Thus, the serious game had a significant positive impact on process model comprehension at follow-up.

Finally, for both research questions, main effect 1 reached significance all the time, indicating that process models are more difficult to comprehend when they are more complex.

### Limitations

First, the external validity and generalizability are limited because only students were examined. Second, to investigate the impact of the serious game on process model comprehension, participants needed to answer ten true or false comprehension questions on five different process models. To ensure comparability of the comprehension questions between studies 1 and 2, different but still similar process models were used (ie, similar process model structure but different element labeling style). Thus, participants in study 2 might have noticed similarities in the process models while answering the comprehension questions and, consequently, answering of respective questions could have been easier. Third, the potential fun factor while playing the serious game may have affected the motivation of participants in answering the comprehension questions. If a task that is associated with some fun factor is completed, then the attitude for the following task is likely to be different and potentially more positive. Accordingly, the motivation of participants of study 1 in the game group could have been different from the control group, resulting in a better comprehension questions score. Fourth, there were participants (n=3) who participated in study 2 but not in study 1. These participants were considered to be a part of the control group instead. Fifth, the better comprehension question scores of the follow-up game group could also be explained by the fact that those participants spent more time working with process models and BPMN in the period between the two measurements (ie, four weeks between study 1 and study 2) during the entry course. Finally, the number of participants in study 1 and study 2 was not the same because there is a typical decrease in the number of students in a course during the term.

### Implications

The provided insights have several implications for practice and research. Particularly noteworthy is the positive immediate and follow-up impact of the serious game in the context of process model comprehension. As known from other domains, this positive effect from serious games may be used to improve formal training in process model comprehension for practitioners or domain experts [35]. Furthermore, existing tools in this context may be enriched with features or aspects of the serious game to improve respective model comprehension.

Moreover, a tool can be developed using the aspects of the serious game to increase general motivation [36]. Practitioners or domain experts can also be supported in their comprehension as well as the creation of process models. Moreover, research on process model comprehension can better investigate the precise impact of specific aspects from a serious game, particularly, which aspects of these game (eg, high score and item crafting) have a beneficial impact and which aspects have an opposite effect [37]. With the use of serious games, another approach can be used to investigate learning success as well as engagement in process model comprehension. Compared to serious games, other game-based principles (eg, gamification and playful interaction) and their impact on process model comprehension can be considered in future research [38]. Finally, research questions may address the impact of a serious game on, for example, cognitive load [39], or if a serious game supports the reduction of the perceived cognitive load while comprehending process models.

### Future Work

In general, based on the insights of study 1 and study 2, the application of the developed serious game had a positive, immediate, and follow-up impact on process model comprehension. Although results look auspicious, additional studies are needed to replicate these findings. The participants of the current study were students taking part in a Business Process Management entry course at a university and studies with other samples are needed to enhance generalization of the results (external validity). For example, it is interesting to investigate the impact of the serious game while including domain experts from different fields (eg, doctors) to investigate whether domain experts are able to learn how to comprehend process models while playing the serious game. Such studies including samples that are not familiar with process modeling should include an assessment of process model comprehension performance at baseline, i. e. before the game group plays the serious game in order to evaluate pre-post changes in the game group vs. control group. The current study included only post- and follow-up assessments, since participants were familiar with business process modeling at least to some extent at the start of the study. To enhance internal validity, a randomized controlled study would be welcome, since the current studies are based on a quasi-experimental design.

The game will also be continuously enriched with new features. These include the introduction of additional BPMN version 2.0 process modeling elements, which has been considered to enable the comprehension of complex or even real-world process models. The focus of the paper at hand was on process model comprehension, and thus we will investigate the impact of the serious game in the context of process modeling as well. Therefore, a feature is currently being implemented that records the steps of process modeling for participants. The recording feature, in turn, will allow us to examine the single steps of process modeling in more detail to enhance our general understanding of working with these models. Finally, support for other process modeling languages (eg, event-driven process chains and Unified Modeling Language activity diagram [40]) will be the subject of future developments.

### Summary

This paper presented the serious game, Tales of a Knightly Process, which introduces the basics of process modeling to foster the comprehension of these models. We also evaluated the immediate and follow-up impact of the serious game on process model comprehension. The results obtained from both studies showed that the serious game had a significant, positive, immediate and follow-up impact on the comprehension of process models. Furthermore, it was observed that process models are increasingly difficult to comprehend with a rising level of model complexity. Altogether, the obtained results highlight the positive impact of the serious game in the field of process model comprehension. With this work, we can therefore confirm and recommend the use of game designs as well as related principles (ie, serious games and gamification) in a nongame context (ie, process model comprehension).

## Abbreviations

BPMN: Business Process Model and Notation
